# Global Transcriptomic Analysis of Inbred Lines Reveal Candidate Genes for Response to Maize Lethal Necrosis

**DOI:** 10.3390/plants14020295

**Published:** 2025-01-20

**Authors:** Ann Murithi, Gayathri Panangipalli, Zhengyu Wen, Michael S. Olsen, Thomas Lübberstedt, Kanwarpal S. Dhugga, Mark Jung

**Affiliations:** 1 Corteva Agriscience, 7000 NW 62nd Ave, Johnston, IA 50131, USA; gayathri.panangipalli@corteva.com (G.P.); mark.t.jung@corteva.com (M.J.); 2 International Maize and Wheat Improvement Center (CIMMYT), Carretera México-Veracruz, Km. 45, Texcoco 56237, Mexico; allen.wen@keygene.com (Z.W.); dhuggaks@gmail.com (K.S.D.); 3 Genetics and Genomics Graduate Program, Iowa State University, 2014 Molecular Building, 2437 Pammel Dr., Ames, IA 5001, USA; 4 Keygene, Inc., 9600 Gudelsky Dr., Rockville, MD 20850, USA; 5 Bayer, Crop Science Division, 800 N. Lindbergh Blvd., St. Louis, MO 63167, USA; michael.olsen@bayer.com; 6 Department of Agronomy, Iowa State University, 716 Farm House Lane, Ames, IA 50011, USA; thomasl@iastate.edu

**Keywords:** maize, viruses, MCMV, SCMV, MLN, gene expression, RNA-Seq, disease resistance

## Abstract

Maize lethal necrosis (MLN) is a significant threat to food security in Sub-Saharan Africa (SSA), with limited commercial inbred lines displaying tolerance. This study analyzed the transcriptomes of four commercially used maize inbred lines and a non-adapted inbred line, all with varying response levels to MLN. RNA-Seq revealed differentially expressed genes in response to infection by maize chlorotic mottle virus (MCMV) and sugarcane mosaic virus (SCMV), the causative agents of MLN. Key findings included the identification of components of the plant innate immune system, such as differentially regulated R genes (mainly LRRs), and activation/deactivation of virus resistance pathways, including RNA interference (RNAi) via *Argonaute* (AGO), *Dicer-like proteins*, and the ubiquitin–proteasome system (UPS) via *RING/U-box* and *ubiquitin ligases*. Genes associated with redox signaling, *WRKY* transcription factors, and cell modification were also differentially expressed. Additionally, the expression of translation initiation and elongation factors, *eIF4E* and *eIF4G*, correlated with the presence of MLN viruses. These findings provide valuable insights into the molecular mechanisms of MLN resistance and highlight potential gene candidates for engineering or selecting MLN-resistant maize germplasm for SSA.

## 1. Introduction

Maize is grown on over 35 million ha and is a staple crop for more than 70 million people in Sub-Saharan Africa (SSA) [1,2,3]. One of the most serious viral diseases is maize lethal necrosis (MLN) [1]. MLN emerged in Kenya in 2011, wreaking havoc on maize production, with estimated losses ranging between 25 and 100% [1,4]. A lack of MLN-resistant hybrids threatens food security [1]. MLN is caused by the synergistic interaction between maize chlorotic mottle virus (MCMV) from the family *Tombusviridae*, genus *Machlomovirus*, and one of several viruses from the family *Potyviridae*, most commonly sugarcane mosaic virus (SCMV) [4,5,6].

Coordinated efforts between the International Maize and Wheat Improvement Centre (CIMMYT), Kenya Agricultural Livestock Research Organization (KALRO), and other organizations to develop MLN-resistant maize varieties proved to be the most economically feasible and environmentally sustainable approach for MLN control [1,4,7]. Lack of resistance in SSA germplasm meant identification of the sources of MLN resistance first and then introgression into the locally adapted hybrids. Association mapping studies and Joint Linkage Association mapping (JLAM) using Drought-Tolerant Maize for Africa (DTMAS), Improved Maize for African Soils (IMAS) panels, and a DH population revealed Quantitative Trait Loci (QTL) associated with MLN resistance on chromosomes 1, 3, 5, 6, 7, and 9 [8,9,10]. Recently, two parallel genome mapping studies using populations derived from crosses with two MLN-resistant sister lines, KS23-5 and KS23-6, revealed a recessively inherited major-effect QTL on chromosome 6, qMLN.06_157 [11]. The donor lines were developed at Kasetsart University in Thailand [12,13]. Other researchers have worked to identify genes scmv1 and scmv2 associated with SCMV resistance through genetic mapping [14,15,16]. Recently, an experiment investigating the role of the translation initiation factor 4E (eIF4E) in resistance to SCMV produced edited eIF4E variants that effectively confer resistance to the virus [17]. High-throughput experimental studies such as transcriptomics can provide insights into the complexities of the biological processes activated in plants in response to various treatments [18]. Transcriptomic studies have previously been reported in maize to evaluate the expression of genes in response to various stresses [19,20,21,22,23]. Expression studies on the response of maize plants infected with SCMV identified an atypical h-type thioredoxin, a candidate gene associated with the Scmv1 QTL on chromosome 6 [24,25,26,27]. A proteomic study in maize identified several differentially abundant proteins (DAPs) in response to MCMV, including two proteins, disulfide isomerase-like protein and ZmPDIL-1 and peroxiredoxin family protein ZmPrx5. Gene-silencing experiments further validated these findings, demonstrating that the absence of these proteins suppressed MCMV, suggesting they act as positive regulators of the virus [28].

Although qMLN.06_157 and the edited eIF4E offer novel sources of resistance to MLN, more sources are crucial to ensure durable disease management to mitigate the risk of pathogen adaptation and resistance breakdown. We, therefore, hypothesize that the response of the maize transcriptome to MLN infection could help reveal the biological processes involved in MLN defense response and possibly identify new gene targets for breeding or gene manipulation. Understanding the mechanisms and, thus, the plant response to the viral infection will facilitate the identification of novel breeding approaches toward MLN resistance. RNA sequencing (RNA-Seq) is a method used to quantify changes in expression levels in the transcriptome in response to external (biotic or abiotic) or internal (growth and development) stimuli [29,30]. The knowledge of the complete transcriptome in a cell is necessary to interpret the functions of genes, reveal the molecular constituents of tissue and cells, understand development, and, in this case, disease response [29]. Consequently, the objectives of this study were to perform an RNA-Seq analysis to (i) analyze transcriptome changes across five tropical maize inbred lines with varying levels of resistance to MLN, (ii) identify potential candidate genes for MLN resistance, and (iii) associate candidate genes to either SCMV or MCMV resistance.

## 2. Results and Discussion

### 2.1. Overview of the RNA-Seq Results

To generate a whole-genome view of changes in the transcriptome of maize plants in response to MLN infection, the expression of mock/null maize samples was compared to MLN-infected plants via high-throughput sequencing. The lines tested were four CIMMYT lines (CMLs) and one line non-adapted to sub-Saharan Africa. Samples infected with MLN were sampled 72 h after inoculation. After low-quality reads and adapter sequence were trimmed, RNA-Seq yielded 33,561,693 to 92,029,813 and 56,165,105 to 114,853,577 clean reads for the mock group and MLN-infected samples, respectively. With a mapping rate >78% in both groups, mapped reads ranged from 27,513,876 to 78,372,589 in the mock group and 46,167,716 to 95,282,527 in the MLN-infected group (Table S1).

After a row-wise normalization of gene expression data in DESeq2, hierarchical clustering was performed to demonstrate technical reproducibility across all samples. This approach grouped samples with similar expression patterns into clusters (Figure S1).

### 2.2. Gene Expression Levels in Response to MLN in Maize

**Figure 1:**
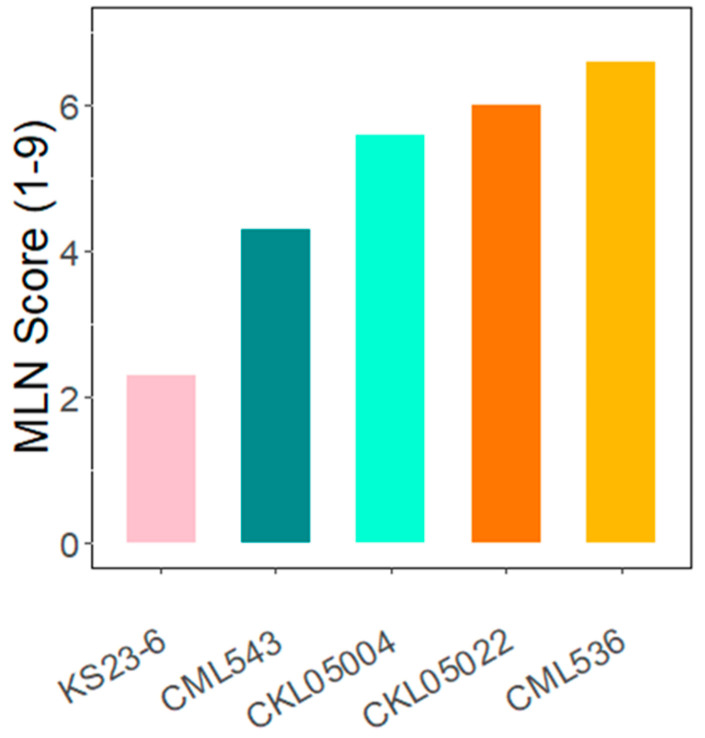
Phenotypic effect of MLN. Average field MLN phenotypic scores of five lines collected in Naivasha, Kenya.

**Figure 2:**
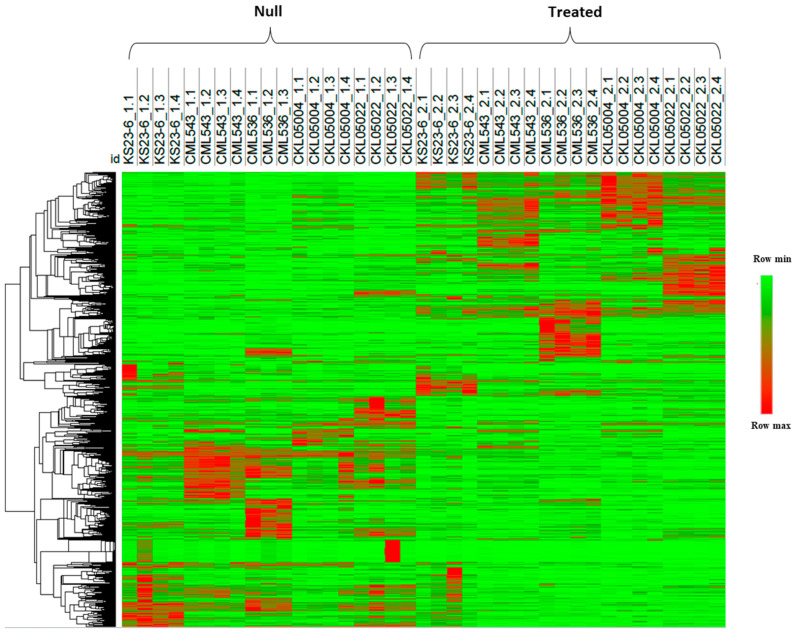
Heatmap clustering of 2503 differentially expressed genes based on their normalized counts. Each row of the heatmap represents the gene count in each genotype before and after inoculation. Red indicates high counts of a gene in a biological replicate, while green indicates lower counts of a gene. “Null” indicates the expression before inoculation, and “treated” indicates expression after MLN inoculation.

In this study, transcriptome analysis of leaves inoculated with MLN revealed differentially expressed gene profiles in different genotypes (Supplementary Tables S1 and S2). From field experiments, the average disease scores of the genotypes based on a rating scale of 1 (tolerant) to 9 (susceptible) ranged from 2.3 in KS23-6 to 6.6 in CML536 (Figure 1). KS23-6 is a non-SSA-adapted donor line from Thailand identified during diversity screening for MLN resistance. All the differentially expressed genes were selected based on a log2foldchange ≥2 or ≤−2 with a *p*-value adjusted to ≤0.05. Cluster analysis of the differentially expressed genes (DEGs) showed that DEGs can be divided into two groups based on the experimental conditions, indicating a significant change in gene expression after MLN (Figure 2). In the null status, genes with high expression showed a marked decrease in expression after inoculation, while genes that were low in the null showed an increased expression in the treated condition.

Cluster analysis of the differentially expressed genes (DEGs) showed the presence of two major groups, divided by gene expression levels in the control group and gene levels in the treated group (Figure 2).

**Figure 3:**
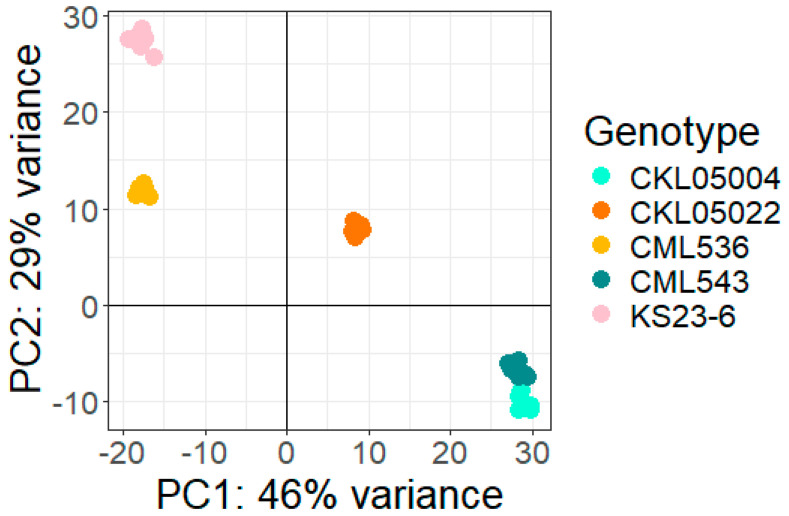
Principal component analysis: Plot generated from normalized gene expression counts of each genotype under MLN.

Principal component analysis (PCA) of the 39,000 gene models based on their normalized counts is shown in Figure 3. PC1 showed that CML543 and CKL05004 were closely related but divergent from the other CMLs. PC1 showed that CML543 and CKL05004 were closely related but divergent from the other CMLs. CKL05002 appeared more different across all the lines. KS23-6 clustered closer to CML536; however, there was a significant difference in the other lines. KS23-6 is a tropical line from Thailand. This proximity may indicate shared ancestry or a common breeding history influencing their genetic composition.

**Figure 4:**
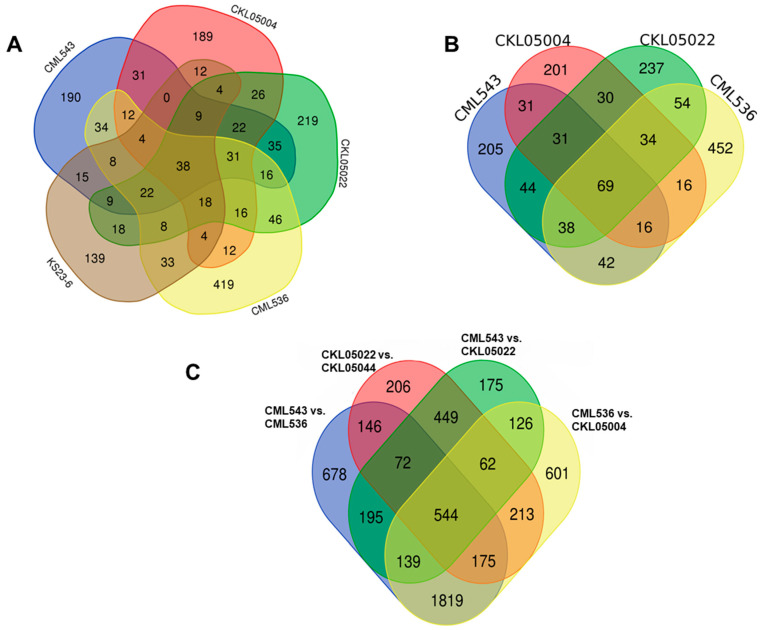
Differentially expressed genes (DEGs). (**A**) Venn diagram of the DEGs in five lines under MLN stress, (**B**) Venn diagram of the DEGs in CIMMYT lines, and (**C**) Venn diagram of the DEGs of the genotype vs. genotype comparison.

Following the variability seen in the PCA plot, differential expression was organized into three comparisons: (1) differential expression between the control and inoculated samples in each line (control vs. MLN treatment), (2) the differential expression after contrasting DEGs across CMLs, and (3) differential expression after contrasting resistant versus susceptible samples. The control vs. treatment experiment indicated the DEGs identified in each line (Table S2). Differential expression of the control versus treatment of ~39,000 maize transcripts that mapped on the five lines in this study revealed 341 DEGs in KS23-6, 721 in CML536, 476 in CML543, 537 in CKL05022, and 428 in CKL05004 (Figure 4A, Table S2). The CML comparisons were designed based on the relatedness using the PCA output, creating four comparisons: (a) CML536 vs. CML543, (b) CML543 vs. CKL05002, (c) CML536 vs. CKL05004, and (d) CKL05004 vs. CKL05022. The relationship between CML543 vs. CKL05004 was not considered because of their relatedness in the PCA, while CML536 vs. CKL05022 was eliminated because there was already more variability detected between CML43 vs. CKL050022 and CML536 vs. CKL05004. In genotype vs. genotype comparisons, there were 3768 DEGs in CML536 vs. CML543 (1820 upregulated and 1948 downregulated), 1762 in CML543 vs. CKL05002 (885 upregulated and 877 downregulated), 3679 in CML536 vs. CKL05004 (1828 upregulated and 1851 downregulated), and 1867 in CKL05004 vs. CKL05022 (926 upregulated and 941 downregulated) (Figure 4B, Table S2). The smallest number of DEGs were detected in the control vs. treatment comparisons, where CML536 had more unique genes than all the other lines (Figure 4A, Table S2). This outcome, where DEGs increase when contrasting two genotypes, corresponds with the work performed by [31]. In the comparison of resistant vs. susceptible samples, there were 3907 DEGs in CML536 vs. KS23-6 (1613 upregulated and 2294 downregulated) and 3851 DEGs in CML543 vs. KS23-6 (1647 upregulated and 2204 downregulated) (Figure 4C, Table S3). CML536 and CML543 were chosen as the susceptible genotypes because, although susceptible, their phenotypic scores differed significantly compared to CKL05004 and CKL05022 (Figure 1). Based on the contrast analysis, upregulated genes in one line were downregulated or had no differential in the contrasted line. For instance, since the contrast was coded (1,0) in CML536 vs. CML543, when a gene was upregulated in CML536, it was either downregulated or undetected in CML543 and vice versa.

### 2.3. Gene Ontology (GO) Enrichment Analysis of Differentially Expressed Genes (DEGs)

**Figure 5:**
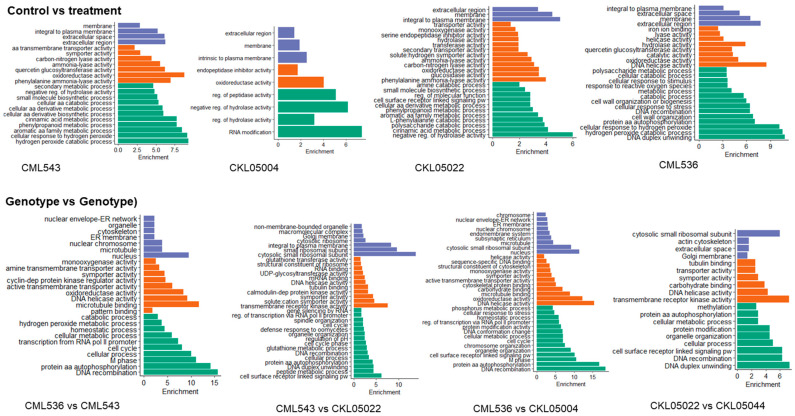
Gene Ontology (GO) enrichment of differentially expressed genes (DEGs) after MLN. A Fisher’s exact test and Bonferroni correction were used to identify the significantly (*p*-value < 0.05) enriched GO terms from the total DEGs across the CIMMYT lines relative to all genes in the maize genome. The first group represents enriched GO terms in each genotype, while the second group represents GO enrichment after contrasting expressions between the lines. The *Y*-axis represents the DEGs’ biological functions, biological process (green), molecular function (orange), and cellular component (purple). The *X*-axis represents the positive values of the estimated *p*-values, calculated as −log10(*p*-value) via GO term analysis.

Functional enrichment analysis was performed to study the DEGs further. We considered three categories of GO enrichment analysis (biological process (BP), molecular function (MF), and cellular component (CC)). Across all the comparisons and lines, GO terms related to stress/defense response were enriched; for instance, in the control vs. treatment group, GO terms significantly enriched in CML543 included the hydrogen peroxide catabolic pathway (GO:0042744) and cellular response to hydrogen peroxide (GO:0070301) (Figure 5; Table S4). CKL05004 showed enrichment in RNA modification (GO:0009451), while CKL05022, among the many GO terms enriched, was cell surface receptor-linked signaling pathways (GO:0007166) (Figure 5; Table S4). CML536 showed enrichment in DNA duplex unwinding (GO:0032508), the hydrogen peroxide catabolic process (GO:0042744), and protein amino acid autophosphorylation (GO:0046777), while in molecular function, DNA helicase activity (GO:0003678) was enriched (Figure 5; Table S3). On the other hand, KS23-6 showed enrichment of secondary metabolic processes, including flavonoids and phenylpropanoid biosynthetic processes, which were also seen in CML543. Across all the lines, oxidoreductase activity (GO:0016709) was enriched in molecular function.

**Figure 6:**
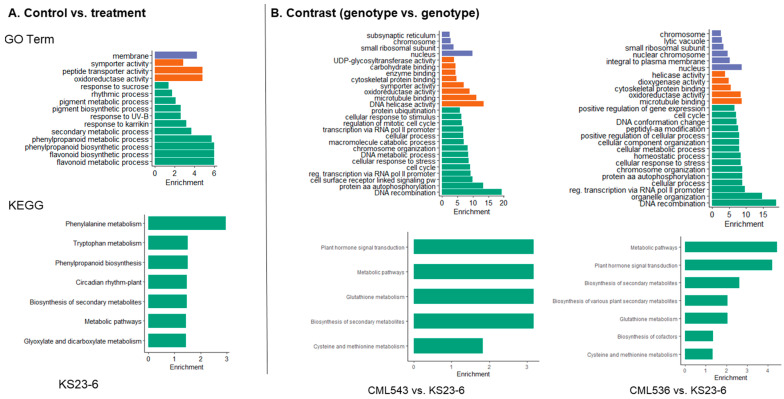
Gene Ontology (GO) and KEGG enrichment of DEGs in KS23-6. Column (**A**) shows the GO terms and KEGG of DEGs in KS23-6 after comparing the control vs. treatment groups, and column (**B**) shows GO terms and KEGG pathways after comparing KS23-6 DEGs to CML543 and CML536. In both, a Fisher’s exact test and Bonferroni correction were used to identify the significantly (*p*-value < 0.05) enriched GO terms and KEGG pathways. In the GO term bar charts, the *Y*-axis represents the DEGs’ biological process (green), molecular function (orange), and cellular component (purple), while the *X*-axis represents the positive values of the estimated *p*-values, calculated as −log10(*p*-value) via Go term analysis.

There was significant enrichment of DNA recombination (GO:0006310) GO terms as well as GO terms associated with the cell cycle (GO:0007049) across all the genotype vs. genotype comparisons. In the molecular function GO term category, DNA helicase activity (GO:0003678) was enriched in all the CML comparisons (Figure 5; Table S5). In all the comparisons without CML536, the cell surface receptor-linked signaling pathway (GO:0007166) was enriched (Figure 5 and Figure 6; Table S5). Transcription from the RNA polymerase II promoter (GO:0006366) and regulation of transcription from the RNA polymerase II promoter (GO:0006357) were enriched in all the comparisons except for CKL05022 vs. CKL05004. More importantly, the term gene-silencing by RNA (GO:0031047) and the defense response to oomycetes (GO:0002229) were enriched in CML543 vs. CKL05022, while the term cellular response to stress was enriched in CML536 vs. CKL05004, CML536 vs. KS23-6, and CML543 vs. KS23-6 (Figure 5 and Figure 6; Table S5).

**Figure 7:**
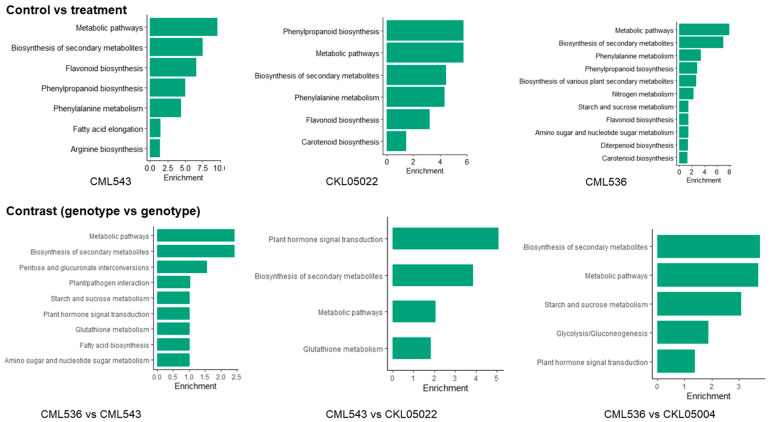
Kyoto Encyclopedia of Genes and Genomes (KEGG) pathway enrichment analyses. A Fisher’s exact test and Bonferroni correction were used to identify the significantly (*p*-value < 0.05) enriched KEGG terms from the total DEGs. The first group represents enriched GO terms in each genotype, while the second group represents GO enrichment after contrasting expressions between the lines. The *Y*-axis represents the KEGG terms. The *X*-axis represents the positive values of the estimated *p*-values, calculated as −log10(*p*-value).

To characterize the complex behavior of the maize transcriptome, all the DEGs were subjected to a KEGG pathway enrichment analysis. Similar to GO enrichment, in KEGG, pathways associated with stress/defense response were enriched. In the control vs. treatment comparison, the biosynthesis of secondary metabolites was central, where biosynthesis and metabolism of various secondary metabolites were indicated across all the CMLs and KS23-6 (Figure 6 and Figure 7; Table S6). Some secondary metabolite pathways observed were flavonoid biosynthesis, phenylpropanoid biosynthesis, carotenoid biosynthesis, and diterpenoid biosynthesis (Figure 7; Table S6). Only one KEGG pathway, Ribosome biogenesis in eukaryotes, was identified in CKL05004 (Table S6). In the genotype vs. genotype comparisons, including the biosynthesis of secondary metabolites, plant hormone signal transduction was also central. In CML536 vs. CML543, CML536 vs. KS23-6, and CML543 vs. KS23-6 comparisons, the pathway involving plant–pathogen interactions was enriched (Figure 6 and Figure 7; Table S6). The glutathione metabolism pathway was enriched only in CML543 vs. CKL05022, CML536 vs. KS23-6, and CML543 vs. KS23-6 comparisons.

### 2.4. Differentially Expressed Genes (DEGs) Involved in Stress-Related Pathways

To understand the key DEGs regulating MLN response, a functional annotation of all the DEGs was performed. Plants combat viral infection by restricting viral replication and movement through defense mechanisms such as immune receptor signaling (innate immunity), gene silencing, protein degradation (autophagy), and the regulation of metabolic components [32,33]. On the other hand, plant susceptibility genes (S-genes) promote establishment by providing the viruses with translation machinery, aiding in forming replication compartments, and facilitating replication and cell-to-cell movement. The comparisons of control vs. treatment and genotype vs. genotype presented DEGs that may play an important role in either positively or negatively influencing the establishment of MCMV or SCMV. Although KS23-6 is the most resistant line, its resistance phenotype is primarily associated with MCMV, while the CMLs, though not significantly resistant, have been linked to resistance against SCMV through various QTL mapping studies, and in some lines like CML543, MCMV-resistance QTLs were identified [34]. This is not surprising, as CMLs are adapted to sub-Saharan Africa, where SCMV is endemic, whereas KS23-6 originates from Thailand, a region where MCMV was reported prior to its first documented occurrence in Kenya and Africa in 2012. Consequently, it is important to recognize that the resistant vs. susceptible comparisons may highlight genes associated with resistance to either MCMV or SCMV. Together, the individual genotype response (control vs. treatment) (Table S2) and the contrast of genotype vs. genotype (Table S3) revealed gene models associated with disease and stress-/defense-related pathways such as the plant’s innate immune system, DNA damage repair, cell wall modifications, redox signaling, phytohormone expression, the ubiquitin–proteasome system (UPS), RNAi silencing, and metabolic disruption. Differential expression of translation initiation factors was also considered as a potential indicator of susceptibility genes.

### 2.5. Innate Plant Immune Response

The pathogen-/microbe-/damage-associated molecular pattern (PAMP/MAMP/DAMP)-triggered immunity (PTI) is the first line of defense against a pathogenic attack, while effector-triggered immunity (ETI), the second line, is triggered by interactions of plant R proteins and pathogen effector proteins [32,35]. The R proteins prevent pathogen movement either by directly or indirectly recognizing the virulence effectors emitted by the pathogen, thus activating immune responses and a cascade of reactions, resulting in the induction of genes associated with defense responses [32,35]. From GO terms, the cell surface receptor-linked signaling pathway, protein amino acid autophosphorylation, cellular response to stress, and cellular response to stimulus were enriched. Many of these receptor kinases, like *leucine-rich repeat* (LRR) protein kinase family protein, were identified in CML536 (Table S2); however, in the genotype vs. genotype comparisons, the number of differentially expressed LRRs was >20 (Table S3). The pattern of expression of the LRR- and NB-ARC-domain-containing disease resistance protein was 50/50 across the CML comparisons, where the number of upregulated and downregulated LRRs was almost equal; for instance, of the 52 LRRs identified in the CML536 vs. CML543 comparison, 25 were downregulated, while the other 27 were upregulated. Similarly, in CML536, of the 16 LRRs identified, 8 were upregulated, and the other 8 were downregulated. On the other hand, of the 43 LRRs identified in the CML536 vs. Ks23-6 comparison, 26 were downregulated, while of the 47 identified in the CML543 vs. KS23-6 comparison, 26 were also downregulated (Table S3). This trend shows that more LRRs were upregulated in KS23-6 compared to the CMLs. Resistance to viruses via LRRs is rare; however, one well-characterized NB-LRR is the tobacco N protein that directly interacts with the helicase domain of the *tobacco mosaic virus* (TMV) replicase to establish resistance [36]. Furthermore, extreme resistance associated with NLR genes has also been characterized in *potato virus X* (PVX) and resistance to certain *soybean mosaic virus* (SMV) strains [37]. Gene models for disease resistance family protein with domain, LRR, CC-NBS-LRR, or dirigent-like protein were also differentially expressed in the genotype vs. genotype comparisons (Table S3). This expression pattern of LRRs and other cell receptor kinases seen here implies a possible resistant vs. susceptible mechanism, where the host produces genes to divert the viruses. In contrast, the viruses may have a stronger mechanism to suppress/bypass the host.

Differential expression of transcription factors was notable across the comparisons, indicating diverse transcriptional regulations in response to MLN. Slavokhotova [38] showed differential expressions of TFFs, including *WRKY*, *NAC*, and *MYB*, in a transcriptomic analysis of cucumber plants infected with cucumber mottle mosaic virus. Similarly, under SCMV, Akbar [39] demonstrated an upregulation of *WRKY*, *AP2*, *NAC, bZIP*, and *bHLH* in the resistance genotype, indicating a possible mechanism for establishing resistance. The distribution and regulation of *WRKY* TF show an upregulation in the susceptible genotypes and a downregulation in the more tolerant genotypes. For instance, all the WRKY identified in three CMLs were upregulated except in CML543 (Table S2). In the CML536 vs. CML543 comparison, *WRKY* was mainly (4 out of 13 were downregulated) upregulated (Table S3). Interestingly, *WRKY* in KS23-6 was upregulated, while the *WRKY* gene models detected in the CML543 vs. KS23-6 comparison were mainly (5 out of 17 were upregulated) downregulated. This comparison shows that nine *WRKY* genes were downregulated in the CML536 vs. CML543 comparison, while 12 were downregulated in the CML543 vs. KS23-6 comparison. This means there was more upregulation of *WRKY* gene models in KS23-6 compared to CML543, while the CML536 vs. CML543 comparison showed more upregulation in CML543. In virus resistance, *WRKY1-WRKY3* are attributed to N-gene-mediated resistance to *tobacco mosaic virus* (TMV), while *WRKY8* functions by restricting the long-distance movement of crucifer-infecting TMV (TM-cg) [40,41]. The expression pattern identified here could indicate a function of *WRKY* TFs in facilitating tolerance or susceptibility to the MLN virus.

#### Redox Signaling

The production of reactive oxygen species (ROS) is one of the first defense responses employed by plants, which can contribute to plants’ defense against viral infections or facilitate viral infection and spread [42,43]. Moderate ROS levels serve as adaptive and defensive signals, whereas excessive ROS can lead to oxidative damage, impair cellular functions, and exacerbate symptom development during viral infections [42]. In all the comparisons, GO terms associated with the hydrogen peroxide catabolic process and oxidoreductase activity were enriched (Figure 5). In CML536, 10 peroxidase genes were identified, where only one was upregulated, while four of the nine identified in CML543 were upregulated (Table S2). Across the lines, more peroxidase gene models were upregulated in CML543 compared to the other CMLs, while CKL05022 and CK05004 showed more upregulation patterns than CML536. In the CML536 vs. CML543 comparison, more peroxidase gene models were shown to be differentially expressed in favor of CML543; however, in the comparison involving KS23-6, all the peroxidases are downregulated, meaning that they were majorly upregulated in KS23-6. This ROS regulation confirms the findings on redox regulation in the study of proteomic changes in maize infected with MCMV [28]. Although their exact functions are not fully understood, Dang demonstrated that ROS response genes, such as *protein disulfide isomerases* (PDIs) and *peroxiredoxin proteins* (Prx), positively regulate MCMV accumulation. Interestingly, the upregulation of peroxidase in KS23-6 and CML543 contradicts these findings; we hypothesize that the presence of SCMV may also influence the regulation of peroxidases. Since the accumulation of ROS is a main symptom of virus infection leading to cell death and necrosis, the expression pattern of peroxidases seen here could explain why the symptoms of MLN are less severe in CML543 than in other CMLs. Furthermore, these results are consistent with Dang’s [28] finding where *2-oxoglutarate (2OG)*- and *Fe (II)-dependent oxygenase superfamily protein*, a component that plays a vital role in the generation of ROS, are upregulated in CML536 (susceptible) and downregulated in KS23-6 and CML543 (tolerant). Here, the expression pattern of these two gene families likely points to their possible involvement in preventing the accumulation of MLN viruses and, more specifically, MCMV.

Conversely, gene models of the *thioredoxin* family protein appear to be mainly upregulated in CML536 and downregulated in KS23-6 (Table S2). The gene, which encodes an atypical *h-type thioredoxin*, was found to be the causal gene for SCMV-resistant QTL, scmv1, on chromosome 6 [24]. The *thioredoxin* family protein is unique to the comparisons with CML536, where five out of seven identified in the CML536 vs. CML543 comparison were upregulated, while eight out of the nine identified in the CML536 vs. CKL05004 comparison were upregulated (Table S3). More importantly, in the CML536 vs. KS23-6 comparison, 8 out of 12 *thioredoxins* were upregulated, while in the CML543 vs. KS23-6 comparison, 6 out of 11 were upregulated. Following the function of *thioredoxin* in SCMV resistance, this regulation indicates it may be related to resistance to SCMV. However, it is worth noting that although the induction of *thioredoxin* is higher in CML536, it is still the most susceptible genotype, implying other possible mechanisms of SCMV that subvert the host.

Another important class of ROS-scavenging protein identified was the *Glutathione S transferase* (GST) family protein identified in CKL05004, where it is upregulated and downregulated in KS23-6 (Table S2). Increased GST enzymatic activity has been linked to SCMV resistance in sorghum, where higher GST levels contribute to stronger, early-stage resistance, while reduced GST activity is associated with susceptibility [44]. Interestingly, GO terms associated with the glutathione metabolic process were enriched in the CML543 vs. CKL05004 comparison (Figure 5). In contrast, the KEGG pathway of Glutathione metabolism was enriched in the CML536 vs. CML543 and CML543 vs. CKL05022 and both the KS23-6 comparisons (Figure 6 and Figure 7). In the CML536 vs. CML543 comparison, GST was mainly downregulated (Table S3). On the other hand, there seems to be a balanced regulation of GST in the CML536 vs. KS23-6 comparison; however, in the CML543 vs. KS23-6 comparison, GST was upregulated (Table S3). More so, in the CML543 vs. CKL05004 comparison, GST was mainly upregulated. The expression of GST showed an upregulation in CML543, followed by CKL05022 and CKL05004, with more downregulation in CML536 and KS23-6. The regulation of these ROS-scavenging proteins (thioredoxin and GST) further confirms that SCMV resistance is majorly associated with the CMLs, while MCMV resistance is stronger in KS23-6.

### 2.6. RNAi and Ubiquitin–Proteasome System (UPS)

To achieve successful infection, viruses hijack the UPS to keep the viral protein stable, while plants utilize the UPS system as a defense mechanism to eliminate viral protein [45]. The *RING/U-box superfamily protein*, along with an F-box protein ubiquitin, is a component of the proteasome ubiquitination complex, which has been shown to interact with begomovirus [46]. In this analysis, the *RING/U-box superfamily protein* gene models were mainly upregulated across the lines, including KS23-6; however, in the CML536 vs. KS23-6 and CML543 vs. KS23-6 comparisons, the RING/U-box gene models were more upregulated in KS23-6 (Tables S2 and S3). On the other hand, 8 out of 26 were downregulated in the CML536 vs. CML543 comparison, indicating a higher upregulation in CML543. This trend was also seen in the CML536 vs. CKL05004 comparison, where more upregulation was seen in CKL05004. A similar expression pattern was also seen in gene models associated with ubiquitination, where they were upregulated across the lines, but more induction was seen in KS23-6 and CML543 in the genotype vs. genotype comparisons (Table S3). Therefore, the trend shows higher induction of RING/U-box and ubiquitin gene models in KS23-6 and CML543 compared to the other CMLs, where CML536 showed a downregulated trend in the genotype vs. genotype comparisons. The role of the UPS system in resistance to MLN viruses has not been established. Still, the differential expression seen here indicates a possible interaction of the viruses with these proteins that either help with their establishment (CML536) or, to a low degree, provide some tolerance (KS23-6/CML543) to the viruses.

Alteration in the expression of gene models associated with the RNA-silencing pathway (RNAi) is another potential antiviral defense mechanism observed here that results in the translational repression of viral RNA [47,48,49]. A gene model involved in gene silencing identified here is the *Argonaute*, which was detected and downregulated in CML543, CML536, and KS23-6 (Table S3). However, the pattern of induction is higher in CKL05004 and CKL05022 than in CML543 and CML536. Alternatively, in the comparisons with KS23-6, *Argonaute* gene models were mainly upregulated in KS23-6 (Table S3). Overall, in relation to the detection, *Argonaute* was mainly downregulated in the CMLs. Consistent with the regulation of *Argonaute*, the *dicer-like protein* was identified and downregulated in the genotype vs. genotype comparisons with CML543. Like the dicer-like protein, gene models associated with *double-stranded RNA-binding protein* (DsRBD protein-related) were detected in the CML543 comparison, where they were downregulated (Table S3). The DsRBD protein-related was also detected in the CML536 vs. CKL05004 comparison, where it was upregulated, indicating a downregulation in CKL05004. To promote their establishment, well adapted plant viruses have evolved mechanisms to produce silencing-suppressor proteins, which can inhibit the host antiviral response based on silencing [50,51]. The potyviral *helper component proteinase* (HCPro) is a well-studied suppressor of antiviral RNA silencing. It performs various functions to inhibit the production of virus-derived siRNAs (vsiRNAs), including binding to ds-RNA, binding to HEN1, inhibiting the activity of HEN1 methyltransferase, interacting with RAV2 to block primary siRNA production, and downregulating RDR6 [49]. From the expression pattern indicated here, especially in CML543, it is possible that the HC-Pro of SCMV plays a major role in downregulating the host silencing machinery to facilitate its colonization, indicating the activation of the host RNA-silencing cascade is important in establishing SCMV.

### 2.7. Cell Cycle Regulation and DNA Damage and Repair

Viruses can alter the host cell cycle to establish conditions that support their replication by disrupting cell cycle checkpoints [52]. Across the genotype vs. genotype comparison, at least one GO term associated with cell cycle, M phase, chromosome organization, DNA recombination, and DNA conformation change was enriched (Figure 5 and Figure S7 and Tables S4 and S5). Including CML536 and the genotype vs. genotype comparisons, the regulation of PIF1 helicase was central (Tables S2 and S3). The *PIF1 helicase* gene models were identified across all the comparisons, where of the 18 identified in the CML536 vs. CML543 comparison, 5 were upregulated, while in the CML536 vs. CKL05004 comparison, of the 22 identified, 19 were upregulated (Table S3). In the CML543 vs. CKL05022 comparison, of the seven helicases, five were upregulated, while in the CKL05022 vs. CKL05004 comparison, all the nine *PIF1 helicases* identified were downregulated (Table S3). Interestingly, in the comparisons with KS23-6, the expression of PIF1 helicase was 50/50, where half of the identified gene models were upregulated, and the other half were downregulated (Table S2). The high differential expression of gene models linked to PIF1 helicases was not clear in regard to SCMV and MCMV, but helicases have been shown to be required by viruses for the transcription of viral mRNAs, translation, disruption of RNA–protein complexes, and packaging of nucleic acids into virions [53]. The *DNA repair (Rad51) family protein, RAD3-like DNA-binding helicase* protein and *RPA70-kDa subunit B* were other DNA repair genes that were identified across the comparisons (Table S3). This pattern of regulation here shows an increased induction of genes associated with damage repair in the more tolerant genotype compared to the susceptible genotype (CML536).

Plant *cyclin* or *cyclin-dependent kinase* CDK members are known for their roles in cell cycle progression, transcriptional regulation, DNA repair, and defense responses [54]. Cui [55] showed that in Arabidopsis thaliana, CDK proteins play a direct role in phosphorylating the C-terminal domain (CTD) of RNA polymerase II and regulating the transcription cycle and are essential for *cauliflower mosaic virus* (CaMV) infection, as the virus utilizes these proteins to activate the transcription of its genes. Across the lines, all gene models for cyclin family protein were downregulated (Table S2). In the CML536 vs. CML543 comparison, cyclin was downregulated, and six cyclin genes identified in the CML543 vs. CKL05022 comparison were upregulated. This indicates the upregulation of CDK in CML543. All the seven cyclin gene models identified in the CML536 vs. CKL05004 comparison were downregulated, indicating an upregulation in CKL05004 (Table S3). In the KS23-6 comparisons, CDK appears to be upregulated in KS23-6 compared to CML543 and CML536. The expression pattern observed here shows an upregulation of CDK gene models in KS23-6 and CML543 compared to CML536, aligning with the increased expression of DNA damage repair genes. This suggests a heightened repair response in the resistant genotypes compared to the susceptible ones. Further research is required to determine the connection between SCMV or MCMV and the suppression of damage repair mechanisms observed in the susceptible genotypes.

### 2.8. Eukaryotic Translation Initiation Factor Expression Under MLN

The translation initiation factors (eIFs) constitute another possible viral resistance system identified in this analysis. The host eIFs assist in the translation of the viral genome, facilitating viral multiplication [56,57,58]. Factors such as *eIF3, eIF4A, eIF4E*, and *eIF5A* are associated with vital processes in vegetative and reproductive growth [59]. In contrast, *eIF1A, eIF2, eIF4*, and *eIF5A* are associated with abiotic and biotic stresses, and eIF4E and eIF4G have been linked to direct interaction with many viruses [60,61]. The eIFs are considered susceptibility (S) genes as they are crucial in viral replication. Both natural and induced recessive resistance associated with mutations in eIF4s (*eIF4E, eIF4G*, and their isoforms) have been exploited in many plants to confer viral resistance [60] (Table 1).

**Figure 8:**
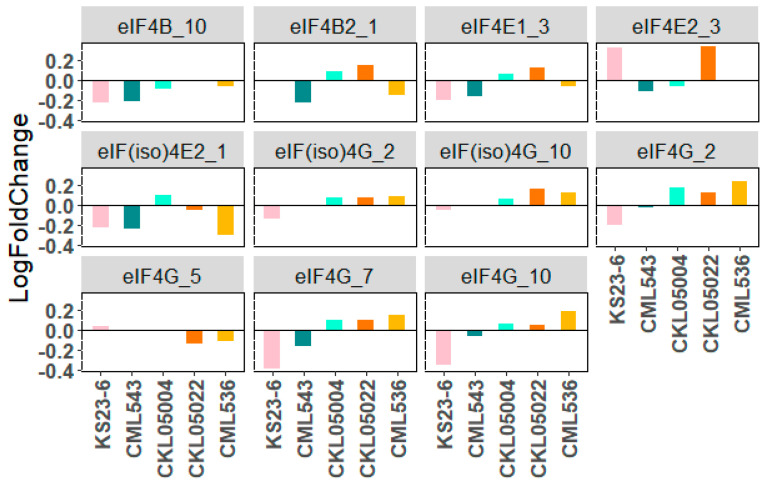
Differential expression of eukaryotic translation initiation factors (eIF4) across the genotypes based on the log2foldchange. The *x*-axis represents the genotypes. The *Y*-axis represents the expression value of each gene within a genotype based on the log2foldchange. (The numbers after the underscores indicate the chromosome locations).

Table S8 shows the expression pattern of different translation initiation factors. We found 32 eIFs, including 1 *eIF1, 6 eIF2s, 8 eIF3s, 11 eIF4s* (4B, 4E, 4G, and their isoforms), 3 *eIF5s, 2 eIF6s, and 1 eIF7*, respectively. Although the expression values are not significant based on the base mean change in gene count between the treatments (log2foldchange > +2 or <−2), there is a detectable differential expression of translation initiation factors correlated to MLN stress (Table S8). For instance, the expression pattern of eIF4E, eIF4E1_3 (Zm00001d041682) and *eIF4E2_3* (Zm00001d041973), and *eIF(iso)4E2_1* (Zm00001d032775) varied across the genotypes (Table 1, Figure 8). In KS23-6, the expression pattern shows an upregulation of *eIF4E1_3* and *eIF(iso)4E2_1* but a downregulation in eIF4E2_3, while CKL05004 portrays an opposite expression pattern to that of KS23-6 (Table 1, Figure 8). In contrast, all the eIF4Es are downregulated in CML543. The locations of *eIF4E1_3* and *eIF4E2_3* on chromosome 3 could be linked to previously identified QTL associated with MLN, mapped on CML543 [8,61]. A recent CRISPR-Cas9 knockout study targeting eIF4E1 and mutations in exon 4 of *eIF4E2* conferred resistance to SCMV, suggesting that the observed downregulation of *eIF4E* in CML543 likely disrupts SCMV growth and replication [17]. Of note, the upregulation of some of eIF4E in KS23-6 further confirms that KS23-6 does not confer resistance to SCMV. Although the differential expression of *eIF(iso)4G_2* (Zm00001d003147) is not indicated in CML543, the expression pattern of the gene shows an expression difference in SSA non-adapted lines (KS23-6), where it is downregulated, and the CMLs, where it is upregulated. The expression of 4Gs in the CML543 is only notable in *eIF4G_2* (Zm00001d006573), *eIF4G_7* (Zm00001d021741), and *eIF4G_10* (Zm00001d025979), where they are downregulated. *eIF4G_2* and *eIF4G_7* show definitive differences in CML543 and KS23-6 with the other CMLs, portraying a clear difference in phenotypic response. This regulation pattern indicates a response of eIF4 to MLN. Given this difference in expression, it would be interesting to examine these genes further as candidate genes for MLN resistance. Understanding the significance of these expression patterns can provide more insight into their relevance to response to viral infection and can be harnessed for managing MLN.

In conclusion, this study demonstrated differences in the responses of the maize inbred lines to MLN, with distinct gene expression patterns observed among the most resistant, moderately resistant, and most susceptible lines. This is incongruent with the established difference in their phenotypic response to MLN, especially between KS23-6 and the CMLs. Our results highlight that KS23-6 resistance is related to MCMV, while the CMLs exhibit resistance to SCMV. This transcriptome analysis also showed the importance of expanding the expression study to more than one line with varying phenotypic responses. The contrast analysis showed that there is a clear transcriptome difference within the CMLs and across them when compared to KS23-6. Gene Ontology and KEGG analysis revealed specific pathways that play a role in stress responses and helped to identify candidate genes that may play an important role in the management of MLN. We identified gene models likely associated with resistance to either MCMV or SCMV, providing insights into the genetic basis of MLN tolerance. Notably, we identified key candidate genes for further investigation, including those involved in innate immune responses (e.g., cell receptor recognition), redox signaling pathways (e.g., thioredoxin, peroxidase, and GST), RNA interference (e.g., *Argonaute, Dicer-like*, and *RNA-binding proteins)*, *WRKY* transcription factors, *ubiquitin E3-ligases*, and *translation initiation factors 4E* and *4G*. Relating these genes to known MLN-resistant QTL can fast-track the process of validating the differentially expressed genes and selecting target genes. Experimental studies on the genes, either through gene knockout of downregulated genes or overexpression, can be employed to understand gene function in response to MLN. Therefore, these genes present promising targets for advancing MLN tolerance through mutational genetics, gene editing, and other biotechnological approaches. While this experiment offers valuable insights into the maize transcriptome at 72 h post-inoculation (hpi), incorporating additional time points would provide a broader understanding of transcriptome changes as viral load increases. Expanding the time points could identify genes activated early versus those expressed later, offering a clearer understanding of genes that positively or negatively influence the MLN viruses.

## 3. Materials and Methods

### 3.1. Plant Materials and Stress Treatment

Four MLN-susceptible CIMMYT lines (CMLs), CML536, CML543, CKL05022, and CKL05004, and one Thailand genotype, KS23-6, were used in this experiment. KS23-6 is known for resistance to MCMV with QTL mapped on chromosome 6, while the CMLs are known for their potential tolerance to SCMV. All the susceptible lines are a set of elite lines used in breeding programs of CIMMYT for hybrid production for SSA. The design followed a randomized block design, where two conditions and four biological replicates were used. All materials were planted in greenhouses, and leaf tissue was sampled twice for RNA extraction. The first tissue sampling was performed immediately before MLN inoculation. Inoculation was then performed on the 3rd and 4th leaves. The second sampling was performed 72 h after inoculation; leaf punches were taken from the 5th and 6th leaves.

MLN inoculum was prepared by combining MCMV and SCMV inoculums. The maize chlorotic mottle virus (MCMV) strain used in this study was isolated by Corteva Agriscience in Hawaii. The sugarcane mosaic virus (SCMV) strain used originated in Ohio and was sourced from USDA at Wooster, OH. MCMV is maintained by serial inoculation in growth chambers, while SCMV is stored in infected tissues at −80 °C at Corteva Agriscience, Johnston, Iowa. The detailed protocol for MLN artificial inoculation has been described in previous studies [9,11,61]. Inoculum was prepared by grinding SCMV and MCMV-infected leaf tissue in 1x phosphate-buffered saline with Tween (PBST) buffer to create a leaf sap. In a Petri dish, MLN inoculum was established by combining/mixing the MCMV and SCMV leaf saps in a ratio of 1:4, respectively. Carborundum was added to facilitate leaf wounding. Plant infection was initiated by gently rubbing the leaf with the sap/carborundum solution.

### 3.2. RNA Sequencing and Data Analysis

To prepare samples for RNA extraction, 8 ¼” leaf punches were taken, frozen immediately on dry ice, and stored at −80 °C before extraction. According to the manufacturer’s protocol, total RNA was isolated from ground-frozen tissue with RNeasy (Qiagen^®^, Germantown, MD, USA). Total RNA was then analyzed for quality and quantity using an Agilent 5300 Bioanalyzer (Agilent Technologies^®^, Santa Clara, CA, USA).

A total of 48 RNA samples (6 genotypes × 4 bio reps × 2 time points) were submitted for library preparation and sequencing at Corteva Agriscience. According to the vendor’s protocol, sequencing libraries were prepared using the Illumina TruSeq stranded mRNA library kit (Illumina Inc., San Diego, CA, USA). The detailed method for RNA-Seq library preparation is described in a study method article [62]. Briefly, mRNAs were isolated via attachment to oligo(dT) beads, fragmented, and reverse-transcribed into cDNA by random hexamer primers with Superscript II reverse transcriptase (Life Technologies, Carlsbad, CA, USA). The resulting cDNAs were end-repaired, 3′-A-tailed, and ligated with Illumina-indexed TruSeq adapters. Ligated cDNA fragments were PCR-amplified with Illumina TruSeq primers, purified with AmpureXP Beads (Beckman Coulter Genomics, Brea, CA, USA), and checked for quality and quantity with the Agilent TapeStation 4200 system with D1000 ScreenTape. Libraries were combined into one sequencing pool and normalized to 2 nM. The pool was denatured according to Illumina sequencing protocols, hybridized, and clustered on an appropriate Illumina flow cell targeting 20M reads per sample. Samples were sequenced using single-read 50bp reads. After sequencing, data were trimmed for quality with a minimum threshold of Q13, and the resulting sequences were split by index identifier.

### 3.3. RNA-Seq Transcript Quantification and Identification of Differentially Expressed Genes (DEGs)

Analysis of the raw reads followed an established RNA-Seq analysis pipeline using the Linux command line. The pipeline begins with preprocessing, where FastQC is used to evaluate the quality of the raw reads (https://www.bioinformatics.babraham.ac.uk/projects/fastqc/). The FastQC filtering identified adapter sequences and assessed the percentage of unknown base sequences and low-quality bases. Trimming of the adapters and sequence repeats was performed using FastX. The downstream analysis was based on quality-controlled clean reads. The publicly available *Zea mays* genome assembly B73 RefGen_v4 (https://www.ncbi.nlm.nih.gov/) was used as the reference genome. Quantification of read abundance was performed using the Salmon tool by aligning the reads to the indexed B73 reference transcriptome [63]. Count data of the reads per gene were generated as transcripts per million (TPM). The count data were subjected to differential expression analysis using DESeq2, a Bioconductor package running on R, version 4.2.1 to identify differentially expressed genes [64]. Differential expression analysis was performed using the general linear model (GLM) implemented in DESeq2. Before fitting and running the model, DESeq2 estimates size factors to normalize the gene counts to account for technical variation, i.e., library size and composition. The dispersion parameter for each gene is also estimated to account for variability between biological replicates using the maximum linear estimation (MLE). The model uses a logarithm link between relative gene abundance and the design matrix:

$${Log}_{2}\left(q_{ij}\right)=\sum_{r}x_{jr}\beta_{ir}$$

DESeq2 integrates the design matrix elements *x_jr_*, of whether sample j is treated, and returns a coefficient *β_ir_*, indicating the overall expression strength of gene *i* as a log2foldchange between the treatment and control. *q_ij_* is the expected true concentration of gene fragments in a sample [64,65].

The Wald test was used for significance testing, and *p*-values that passed independent filtering were adjusted for multiple testing using the Benjamini and Hochberg False Discovery Rate (FDR) procedure [64]. Differential expression results were filtered to select differentially expressed genes (DEGs), and only genes with a 2-fold change in expression level and ≤5% FDR were selected. Clustering tools, heatmaps, and principal component analysis (PCA) embedded in the DESeq2 package were used to generate PCA plots and heatmaps based on transformed and normalized gene expression counts. An online Venn generator (https://bioinformatics.psb.ugent.be/webtools/Venn/) was used to identify common and unique DEGs across genotypes. K-mean clusters were derived via normalized gene counts of the DEGs using the K-means clustering tool provided by Morpheus (https://software.broadinstitute.org/morpheus/).

### 3.4. DEG Functional Annotation and Enrichment Analysis

To perform the functional annotation of the genes, Gene Ontology, KEGG, and manual annotation were performed using public databases. Using AgriGO [66], the DEGs were subjected to Gene Ontology (GO) analysis for biological process (BP), molecular function (MF), and cellular composition (CC). Maize AGPv4 provided by the software was used as the annotation reference. Fisher’s exact test with multi-test adjustment was used, and GO terms were selected based on *p_adj_* < 0.05. Further, a Kyoto Encyclopedia of Genes and Genomes (KEGG) analysis was performed in ShinyGO (https://doi.org/10.1093/bioinformatics/btz931) to identify enriched pathways. To identify gene annotations associated with the IDs of the DEGs, a maize gene ID list (based on B73 v4 gene models) with functional annotations sourced from Ensemble Plants biomart (https://plants.ensembl.org) was used to match the DEGs’ gene IDs to protein functions.

## Figures and Tables

**Table 1:** Differential expression of eukaryotic initiation factor 4s (eIF4s) and their isoforms after MLN infection in each genotype. The color show a gradient of downregulation (negative) and upregulation (positive), where red is downregulated and Green is upregulated.

Gene_Name	Gene_ID	KS23	CML543	CKL05004	CKL05022	CML536	Protein Name	Chr
eIF4B_10	Zm00001d025692	−0.22	−0.21	−0.09	−0.01	−0.07	Eukaryotic translation initiation factor 4B1	Chr10
eIF4B2_1	Zm00001d003288	−0.01	−0.22	0.08	0.14	−0.15	Eukaryotic translation initiation factor 4B1	Chr2
eIF4E1_3	Zm00001d041682	−0.20	−0.16	0.06	0.12	−0.06	Eukaryotic translation initiation factor 4E	Chr3
eIF4E2_3	Zm00001d041973	0.32	−0.12	−0.06	0.34	−0.01	Eukaryotic translation initiation factor 4E	Chr3
eIF(iso)4E2_1	Zm00001d032775	−0.23	−0.24	0.10	−0.05	−0.30	Eukaryotic initiation factor 4E protein	Chr1
eIF4G_2	Zm00001d006573	−0.21	−0.02	0.17	0.12	0.24	Eukaryotic translation initiation factor 4G	Chr2
eIF4G_5	Zm00001d017310	0.03	0.00	−0.02	−0.14	−0.12	Eukaryotic translation initiation factor 4G	Chr5
eIF4G_7	Zm00001d021741	−0.38	−0.17	0.09	0.10	0.15	Eukaryotic translation initiation factor 4G	Chr7
eIF4G_10	Zm00001d025979	−0.36	−0.07	0.07	0.04	0.18	Eukaryotic translation initiation factor 4G	Chr10
eIF(Iso)4G_2	Zm00001d003147	−0.14	0.00	0.07	0.07	0.08	MIF4G domain-containing protein	Chr2
eIF(Iso)4G_10	Zm00001d025777	−0.06	0.00	0.06	0.16	0.12	MIF4G domain-containing protein	Chr10

## Data Availability

The authors confirm that the raw data supporting the findings in this study will be accessible once the permissions for public sharing have been obtained. Meanwhile, the raw outputs from the read quantification and DESeq2 analysis are available at the following GitHub link: https://github.com/AnnM-511/Transcriptome_profiling_6maize_inbred_lines.

## Supplementary Materials

The following supporting information can be downloaded at https://www.mdpi.com/article/10.3390/plants14020295/s1, Table S1: Details of data generated by sequencing across all the biological replicates; Table S2: Differentially expressed genes (DEGs) of each line in control vs. treatment analysis; Table S3: Differentially expressed genes (DEGs) of each genotype vs. genotype comparison; Table S4: GO terms associated with differentially expressed genes in each line from control vs. treatment analysis; Table S5: GO terms associated with differentially expressed genes in genotype vs. genotype comparisons; Table S6: KEGG pathways associated with differentially expressed genes in each line from control vs. treatment analysis; Table S7: KEGG pathways associated with differentially expressed genes of genotype vs. genotype comparisons; Table S8: Differential expression of eukaryotic translation initiation factors (eIFs) across genotypes based on log2foldchange; Figure S1: Heatmap clustering of all biological replicates based on their normalized gene expression counts across all six lines. Red indicates a higher similarity index between biological reps, while blue indicates a lower similarity index, where 1 represents 4 of the null replicates and 2 represents 4 of the treated replicates.
