# Supplementary figures and images for: Global Transcriptomic Analysis of Inbred Lines Reveal Candidate Genes for Response to Maize Lethal Necrosis
Source: Plants (Basel). 2025 Jan 20;14(2):295. doi: 10.3390/plants14020295 (PMC11768128; doi:10.3390/plants14020295)

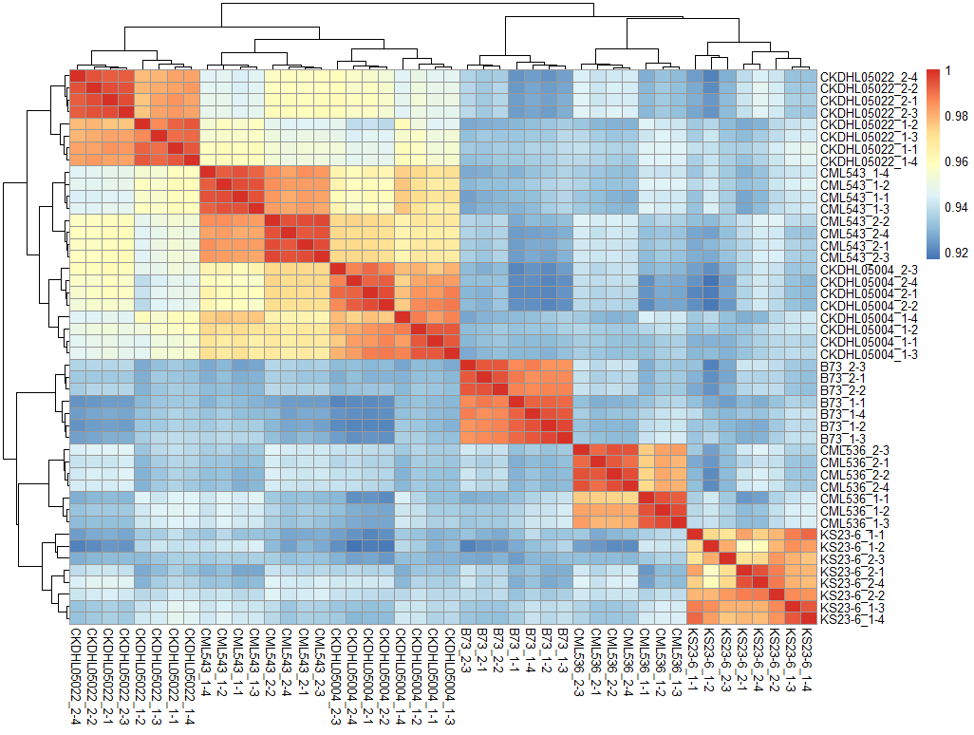

Supplement: Supplementary file 1 [file plants-14-00295-s001.zip › Figure S1.png]
